# Dual-Modal Approach for Ship Detection: Fusing Synthetic Aperture Radar and Optical Satellite Imagery

**DOI:** 10.3390/s25020329

**Published:** 2025-01-08

**Authors:** Mahmoud Ahmed, Naser El-Sheimy, Henry Leung

**Affiliations:** 1 Department of Electrical and Software Engineering, University of Calgary, Calgary, AB T2N 1N4, Canada; leungh@ucalgary.ca; 2 Department of Geomatics Engineering, University of Calgary, Calgary, AB T2N 1N4, Canada; elsheimy@ucalgary.ca

**Keywords:** synthetic aperture radar (SAR), multi-modal fusion, detection transformer

## Abstract

The fusion of synthetic aperture radar (SAR) and optical satellite imagery poses significant challenges for ship detection due to the distinct characteristics and noise profiles of each modality. Optical imagery provides high-resolution information but struggles in adverse weather and low-light conditions, reducing its reliability for maritime applications. In contrast, SAR imagery excels in these scenarios but is prone to noise and clutter, complicating vessel detection. Existing research on SAR and optical image fusion often fails to effectively leverage their complementary strengths, resulting in suboptimal detection outcomes. This research presents a novel fusion framework designed to enhance ship detection by integrating SAR and optical imagery. This framework incorporates a detection system for optical images that utilizes Contrast Limited Adaptive Histogram Equalization (CLAHE) in combination with the YOLOv7 model to improve accuracy and processing speed. For SAR images, a customized Detection Transformer model, SAR-EDT, integrates advanced denoising algorithms and optimized pooling configurations. A fusion module evaluates the overlaps of detected bounding boxes based on intersection over union (IoU) metrics. Fused detections are generated by averaging confidence scores and recalculating bounding box dimensions, followed by robust postprocessing to eliminate duplicates. The proposed framework significantly improves ship detection accuracy across various scenarios.

## 1. Introduction

Optical and synthetic aperture radar (SAR) image fusion for ship detection and recognition has become a crucial area of research due to the complementary advantages of these sensors. While single-source ship detection methods, such as optical-based visual saliency and SAR-based texture analysis, are well established, they are often constrained by the inherent characteristics of the sensor, limiting their ability to fully exploit the benefits of integrating data from multiple sources. This results in partial information loss and limitations in feature representation, which can hinder overall detection performance, especially in challenging environmental conditions like low light, cloud cover, and varying sea states [1].

Over the past few decades, researchers have explored various fusion techniques to combine data from optical and SAR sensors. The most common methods are categorized into pixel-level, feature-level, and decision-level fusion [2]. Pixel-level fusion combines raw pixel data from both optical and SAR images, preserving spectral and textural information, but it requires high-precision sensor registration, which can be difficult to achieve in dynamic environments. Feature-level fusion, however, extracts and combines features from registered optical and SAR images, providing a more flexible and robust approach that supports real-time processing. This method has become increasingly popular due to its ability to handle registration errors and its effectiveness in integrating data from multiple sensors. Decision-level fusion combines the results from individual sensors based on their confidence levels, producing more reliable detection outcomes, particularly in complex scenarios where sensor performance is degraded by environmental factors such as varying illumination, sea state, and atmospheric conditions [3].

Recent advancements in multisource fusion for ship detection involve using deep learning algorithms like YOLO (You Only Look Once) and Faster R-CNN on both optical and SAR images [4]. These algorithms are adaptable to multisource fusion, allowing them to process large volumes of data efficiently while handling complex environments [5]. YOLO, for example, has been widely applied to both optical and SAR ship detection and has evolved from YOLOv1 to YOLOv8, continuously addressing various detection challenges [6]. Innovations such as RetinaNet’s Focal Loss further improve detection accuracy by focusing on difficult samples, but challenges with hyperparameter tuning and computational efficiency remain [7].

Transformer-based models, including the Detection Transformer (DETR), have gained considerable attention for ship detection tasks in multisource fusion scenarios [8]. The DETR, in particular, treats detection as a set prediction problem, eliminating the need for traditional postprocessing steps like non-maximum suppression (NMS) and using anchor boxes. However, the DETR’s high computational demands and slow convergence time can limit its practicality, especially in resource-constrained environments or when detecting small objects [5]. Other Transformer-based models, such as Swin Transformers and Pyramid Vision Transformers (PVTs), have further optimized computational performance through localized self-attention and spatial reduction techniques, making them more suitable for multisource fusion tasks [9,10].

Hybrid methods that combine SAR and optical images using Generative Adversarial Networks (GANs) to convert SAR images into optical-like images have also been explored [11]. These approaches aim to improve fusion quality by mitigating issues such as speckle noise and spectral distortions inherent in SAR imagery. Paired feature fusion methods, which process both optical and SAR images simultaneously to extract complementary features, have shown promise in enhancing ship detection performance. These methods often employ advanced architectures like multiscale feature fusion and channel attention mechanisms, leading to fused features that offer a more comprehensive view of the target [12].

Additionally, self-supervised learning techniques, such as contrastive learning and generative models, enable unified feature extraction from optical and SAR images. These approaches reduce the reliance on labeled data and enhance detection in complex conditions such as cloud cover, shadows, and dynamic sea state changes [13].

Moreover, combining multisource fusion with techniques like using graph neural networks (GNNs) helps capture spatial relationships, leading to improved feature representation and enhanced detection robustness in challenging maritime environments [14].

Advancements in sensor technology, including the development of higher-resolution optical sensors, more sophisticated SAR imaging techniques, and multispectral sensors, have significantly improved the capabilities of multisource data fusion. The integration of high-resolution optical images with detailed SAR data allows for the extraction of richer, more accurate features, improving the detection and recognition of ships while distinguishing them from background noise and small, irregular targets. This significantly enhances detection accuracy and reduces false positives, which is critical in maritime surveillance

Finally, the fusion of optical and SAR images for ship detection and recognition has evolved from traditional methods to deep learning-based approaches that can effectively leverage multisource data across diverse environmental conditions. The integration of these sensors offers substantial benefits, including enhanced detection accuracy, greater resilience to environmental challenges, reduced false positives, and improved robustness in complex scenarios. However, challenges remain, particularly in resource-constrained environments where computational resources are limited. Ongoing advancements in sensor technology, deep learning algorithms, self-supervised learning, and multisource data fusion techniques are crucial to overcoming these challenges and fully realizing the potential of multisource data for ship detection and recognition in optical and SAR images.

### Contributions

The contribution of this research is the development of a novel fusion framework that integrates optical and SAR imagery for enhanced ship detection in complex maritime environments. This framework leverages the complementary strengths of optical and SAR data, addressing the inherent limitations of each modality, such as the sensitivity of optical imagery to adverse weather and low-light conditions, and the noise and clutter challenges of SAR imagery. The key innovations include the following:

- Enhanced Optical Detection

The use of CLAHE combined with the YOLOv7 model for optical imagery significantly improves detection accuracy and processing speed, particularly in challenging conditions.

- Customized SAR Detection

The development of the SAR-EDT model, a specialized DETR, incorporates advanced denoising algorithms and optimized pooling configurations to handle the noise and clutter specific to SAR images, ensuring robust ship detection.

- Model-Driven Fusion Approach

The fusion module effectively combines the detection outputs from both optical and SAR modalities by evaluating spatial overlap through intersection over union (IoU) and merging bounding boxes based on their confidence scores. This fusion methodology improves detection reliability and reduces duplicate detections.

Overall, this research introduces a comprehensive and efficient approach for integrating SAR and optical imagery, offering substantial improvements in ship detection performance, especially under difficult environmental conditions. The proposed framework holds promise for real-time applications in maritime surveillance and security, with future work aimed at further optimizing computational efficiency and expanding its applicability.

## 2. Materials and Methods

**Figure 1:**
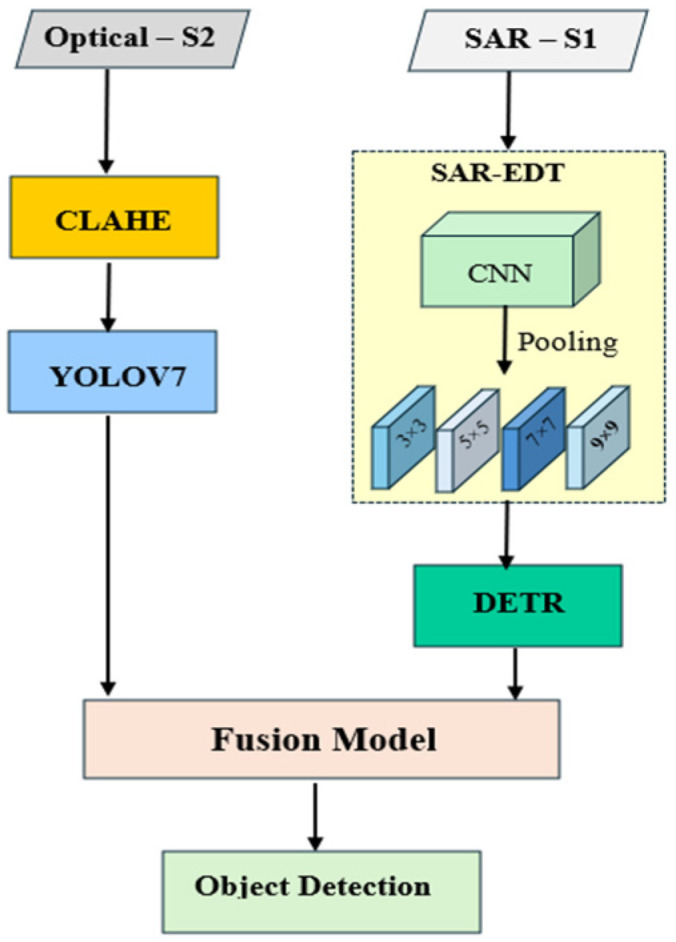
Flowchart of proposed ship detection in SAR and optical imagery.

This research proposes a fusion-based framework designed to enhance object detection accuracy by integrating optical and SAR satellite imagery. The methodology consists of three main components: optical image detection, SAR image detection, and the fusion of detection outputs, as illustrated in Figure 1.

In the optical object detection module, CLAHE is applied to improve contrast and enhance visual details in optical images. The YOLOv7 model, known for its high speed and precision, is employed for detecting objects in enhanced images. This module is optimized to handle variable illumination and resolution characteristics often encountered in optical satellite imagery.

For SAR imagery, this study introduces a customized DETR model termed SAR-EDT. This model incorporates advanced denoising algorithms and optimized pooling configurations, specifically designed to address the unique noise, clutter, and scattering properties of SAR data. A specialized CNN is integrated to predict optimal pooling parameters dynamically. The CNN leverages evaluation metrics such as LSF, PSNR, and F1 score, ensuring robust adaptability to diverse environmental and imaging conditions.

The fusion module combines the detection outputs from the optical and SAR image modules through a model-driven fusion approach. This process evaluates the spatial overlap of detected bounding boxes using the intersection over union (IoU) metric. Bounding boxes with an IoU exceeding a predefined threshold are merged. The fusion process calculates the central coordinates and average dimensions of overlapping bounding boxes to generate unified fused detections. To improve detection reliability, the confidence scores of the fused bounding boxes are averaged, providing a more accurate representation of the likelihood of object presence. Postprocessing techniques are applied to identify and remove duplicate detections by analyzing the centroids of fused bounding boxes, retaining only the detections with the highest confidence scores.

### 2.1. YOLOv7 with Adaptive Contrast Enhancement (YOLOv7-ACE): Framework for Optical Ship Detection

The methodology for YOLOv7-ACE presents an integrated framework combining YOLOv7 with CLAHE. This approach is designed to improve object detection performance, especially in complex imaging environments, by enhancing feature visibility and detection accuracy. The YOLOv7-ACE framework capitalizes on the advanced architecture of YOLOv7, particularly its Enhanced-ELAN (E-ELAN) module, alongside the adaptive contrast enhancement capabilities of CLAHE. This combination helps the system adapt to diverse lighting conditions, improving object detection reliability in various real-world scenarios.

#### 2.1.1. Contrast Limited Adaptive Histogram Equalization (CLAHE)

Ship detection in optical images is challenged by the type of scene—open ocean versus mixed sea–land nearshore backgrounds. In open ocean scenes, ships contrast with the ocean due to differences in color and texture, but detection can be hindered by sea clutter, cloud occlusion, and lighting variations. Nearshore scenes add complexity, as ships can resemble onshore objects like buildings or containers, which increases false alarms and complicates detection [15]. To improve image quality and mitigate environmental effects, such as cloud cover and fog, preprocessing is essential. However, overly simplistic methods can lose critical details in these complex scenes [16].

CLAHE is used in this study to improve contrast in localized regions of the image. Unlike traditional global histogram equalization, which applies a uniform transformation to the entire image [17], CLAHE adapts the enhancement in small, localized regions, or tiles, of the image. This method improves local contrast and preserves image details without amplifying noise, which is especially important for ship detection in varying maritime environments.

The CLAHE process encompasses several critical steps. First, the input image is divided into non-overlapping or overlapping regions known as tiles. Each tile is processed independently to enhance local contrast, allowing for a more tailored enhancement compared to global methods.

For each tile T, the histogram H(r) representing the distribution of pixel intensities is computed as follows:

(1) $$H(r)=\sum_{(x,y)\in T}\;I(x,y)\;\mathrm{for}\;r\in [0,L-1]$$

where I(x,y) denotes the intensity of the pixel at coordinates (x,y) within the tile, and L is the total number of intensity levels.

The cumulative distribution function for the histogram is determined by the following:

(2) $$CDF(r)=\sum_{r^{\prime }=0}^{r}\;H\left(r^{\prime }\right)$$

This function allows for the mapping of pixel intensities to their corresponding probabilities. To prevent the excessive amplification of noise in homogeneous regions, a contrast limiting step is introduced. A predefined clipping threshold c is established, and if the histogram value exceeds this limit, it is modified as follows:

(3) $$H^{\prime }(r)=\left\{\begin{array}{ll} H(r) & \mathrm{if}\;H(r)\leq c\\ c+\frac{H(r)-c}{\sum_{r^{\prime }=0}^{L}\;\;H\left(r^{\prime }\right)-c}\cdot c & \mathrm{if}\;H(r)>c \end{array}\right.$$

This adjustment redistributes the clipped histogram values uniformly while preserving essential image features.

The equalized histogram $H_{eq}(r)$ is calculated based on the modified histogram and the *CDF*:

(4) $$H_{eq}(r)=\mathrm{round}\;(CDF(r)\cdot \frac{N}{L-1})$$

where *N* represents the total number of pixels within the tile. To ensure smooth transitions between adjacent tiles, bilinear interpolation is applied. The intensity of a pixel at position (*x*,*y*) is computed as follows:

(5) $$I^{\prime }(x,y)=\alpha I_{TL}+\beta I_{TR}+\gamma I_{BL}+\delta I_{BR}$$

where $I_{TL},I_{TR},I_{BL},I_{BR}$ are the intensities of the neighboring pixels, and α,β,γ,δ are the weights based on the distances to those neighboring pixels.

The enhanced image $I^{\prime }$ is constructed by combining the processed tiles with smooth transitions between them, resulting in improved contrast while preserving fine details in both dark and bright regions. CLAHE effectively enhances image contrast while mitigating noise effects, making it a valuable tool for optical imaging applications, including ship detection in maritime surveillance.

#### 2.1.2. YOLOv7 Architecture with Enhanced-ELAN (E-ELAN)

The YOLOv7 model represents a significant evolution in the YOLO series, standing out with its exceptional speed and accuracy, making it a prime choice for ship detection in optical imagery. The YOLOv7 model was designed for real-time video surveillance; it has demonstrated remarkable efficiency in complex maritime environments [18], highlighting its potential for optical image-based applications. This model incorporates key architectural innovations, including the Enhanced-ELAN (E-ELAN) module, which greatly improves feature learning. Through advanced techniques like expansion, shuffling, and cardinality merging, YOLOv7 enhances its ability to detect diverse and complex patterns, ensuring precise detections even under challenging conditions.

**Figure 2:**
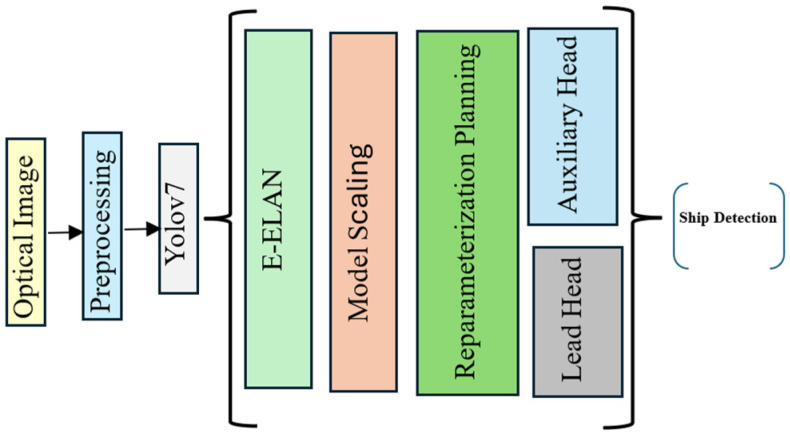
Architecture of Yolov7 for ship detection from optical satellite images.

As illustrated in Figure 2, the workflow starts with preprocessing optical images, which are then passed into the YOLOv7 architecture. The E-ELAN module plays a central role by allowing computational blocks to capture and refine distinct feature sets, enabling the model to learn intricate patterns effectively. This results in enriched feature representations, critical for ship detection. The model also employs a novel reparameterization planning and model scaling approach, ensuring that it adapts efficiently to varying computational resources without compromising accuracy or structural integrity. This flexibility allows YOLOv7 to maintain high performance across different configurations.

Furthermore, Figure 2 highlights how YOLOv7 integrates auxiliary and lead detection heads, combining their outputs to perform accurate ship detection. This dual-head approach enables robust predictions, crucial for applications like maritime surveillance. Leveraging its success in video-based detection, YOLOv7 proves to be a highly capable solution for optical image-based ship detection, offering a blend of speed, accuracy, and architectural robustness to address the challenges of complex object detection tasks in maritime environments.

### 2.2. SAR-EDT: Improving Ship Detection in Complex Maritime Environments with Optimized Pooling Strategies

The effective preprocessing of synthetic aperture radar (SAR) images is crucial for improving feature representation while addressing issues such as speckle noise, clutter, and geometric distortions, which can impair detection accuracy. These issues are especially challenging in maritime environments, where ships often exhibit significant variations within the same class, and complex background noise complicates detection. The SAR-EDT model incorporates an advanced pooling module designed to optimize ship detection in such challenging conditions [19]. This model combines both max pooling and median pooling strategies, using kernel sizes of 3, 5, 7, and 9, to achieve a balance between reducing noise and preserving critical image features.

Max pooling proves beneficial in enhancing the visibility of key ship structures, enabling the model to distinguish these features more effectively from cluttered backgrounds. Median pooling excels at minimizing noise while retaining fine edges, which are vital for accurately defining ship boundaries. The selection of pooling configurations is guided by metrics such as LSF, PSNR, and F1 score, ensuring that the chosen pooling method reduces noise and maintains high image quality.

Comprehensive experiments utilizing ship datasets from SAR satellites, including Gaofen-3 and Sentinel-1, demonstrate the SAR-EDT model’s effectiveness, particularly in challenging conditions where initial detections may be less reliable. These datasets, which offer diverse polarization options (VV and VH), enhance the model’s robustness across varying environmental circumstances. The design of the pooling module significantly boosts detection accuracy, highlighting its essential role in SAR image analysis.

In addition, a CNN was developed and trained using labeled data that incorporates LSF, PSNR, and F1 scores to predict optimal pooling parameters. By utilizing these metrics as input features and target labels, the CNN effectively learns to recommend the best pooling configurations to enhance ship detection in SAR images. This integrated approach guarantees that the selected pooling parameters maintain a delicate equilibrium between edge sharpness, image quality, and overall detection accuracy, thereby refining the ship detection process with the SAR-EDT model.

The combination of rigorous evaluation metrics and innovative pooling strategies not only enhances the model’s performance but also contributes to the overarching goal of improving maritime surveillance and safety. By enabling more accurate and reliable ship detection, this approach has significant implications for real-world applications, including enhancing navigation safety, supporting search and rescue operations, and facilitating maritime law enforcement.

#### 2.2.1. CNN Architecture for Pooling Parameter Estimation

To improve SAR image preprocessing for ship detection, a CNN model is designed to effectively capture the key features of the input data. This model will be trained using supervised learning with labeled SAR images and their corresponding LSF and PSNR values and F1 scores for optimal pooling parameters.

The CNN architecture is designed to process SAR images for ship detection by learning local patterns and extracting important features. The core of the model consists of convolutional layers that apply filters to images, extracting vital features needed for effective detection. The choice of filters and kernel sizes will be optimized through experiments to ensure the model best captures the unique characteristics of SAR images. To enhance the model’s ability to learn complex relationships, the Rectified Linear Unit (ReLU) activation function will be used. After convolutional layers, pooling layers will downsample the feature maps, keeping only the most relevant features while reducing computational complexity. Finally, fully connected layers will map the learned features to the target outputs, predicting the best kernel size and pooling type.

Choosing the right pooling method and kernel size is crucial for improving ship detection accuracy in SAR images. The process of selecting the optimal pooling parameters involves several steps:

1. Evaluation of F1 Scores: F1 scores are evaluated for each pooling type and kernel size configuration to measure detection accuracy comprehensively. The configuration with the highest F1 score is determined as follows:

(6) $${F1\_score}_{max}=max\;({F1\_score}_{i})$$

2. Integration of LSF and PSNR Metrics: Edge sharpness and image quality are assessed across different pooling configurations to make informed decisions regarding the optimal pooling parameters for preprocessing SAR images. This methodical approach ensures that processed images retain sharp edges and high fidelity, improving the efficacy of the SAR-EDT model. The configuration with the lowest LSF and the highest F1 score is selected.

(7) $${LSF}_{min}=min\;\left({LSF}_{i}|F1\_{score}_{i}=(F1\_{score}_{max}\right)$$

3. Handling Multiple Configurations: If multiple configurations have the same F1 score and LSF, the configuration with the highest PSNR is selected:

(8) $${PSNR}_{max}=max\;\left({PSNR}_{i}|{LSF}_{i}={LSF}_{min},\;F1-{score}_{i}=F1-{score}_{max}\right)$$

4. Implementation of Labeling Steps: Once the optimal pooling parameters are determined, they will be applied during the labeling process. These configurations ensure that the model is trained with the best preprocessing settings for improved ship detection.

After training, the CNN model will predict the optimal pooling parameters for new SAR images based on their LSF values. The model will select the best kernel size (3 × 3, 5 × 5, 7 × 7, or 9 × 9) and choose either ‘max’ or ‘median’ pooling. These predictions will guide the preprocessing phase for SAR images, enhancing detection accuracy and overall performance in complex maritime environments.

#### 2.2.2. Detection Transformer Module (DETR)

Ship detection in SAR imagery is challenging due to small target sizes, cluttered backgrounds, and environmental variations. RT-DETR outperforms YOLO models by leveraging its advanced backbone for enhanced feature extraction and contextual understanding, excelling in accuracy and reliability for precision-demanding tasks. In contrast, YOLO models like YOLOv5s, YOLOv5m, YOLOv7, and YOLOv8 prioritize faster inference and lower complexity but struggle with lower recall and precision for small ships. While RT-DETR’s larger parameter count and slower inference limit real-time use, its superior detection capabilities make it ideal for applications requiring high accuracy [20].

The DETR model enhances the original architecture by incorporating the DC5-R101 backbone, significantly improving feature extraction and contextual awareness. This integration effectively addresses common challenges in object detection, such as variations in object scales, complex shapes, and crowded scenes. In the DETR, input embeddings pass through a Transformer encoder–decoder, generating output embeddings that are fed into a classifier feed-forward network (FFN) and a bounding box FFN to yield final predictions. The architecture starts with an input image (*x*) enhanced by positional encodings *PE*(*x*) to accurately identify object locations, applied to both the image and input feature maps for better spatial understanding. The positional encodings for a two-dimensional position (*i*, *j*) are defined as follows:

(9) $$PE\left(i,j\right)=[\sin\;\left(i/10000^{\wedge }\left(2^{⋆}d/emb\_dim\right)\right),\cos\;\left(i/10000^{\wedge }\left(2^{⋆}d/emb\_dim\right)\right)\;\;\;\;\;\sin\;\left(j/10000^{\wedge }\left(2^{⋆}d/emb\_dim\right)\right),\cos\;\left(j/10000^{\wedge }\left(2^{⋆}d/emb\_dim\right)\right)]\;,$$

The DC5-R101 backbone modifies the receptive field through adjustments in dilation and stride, improving feature map output, represented by the following:

(10) $$H_{backbone}=DC5-R101\left(PE\left(x\right)\right),$$

This backbone incurs higher computational costs due to intensive self-attention mechanisms. The backbone’s dilation rate, particularly a rate of 2, expands the receptive field:

*DC*5*Y*, *d* = *ConvY*, 5 × 5, *dilation* = *d* (11)

The architecture also includes a Transformer encoder with self-attention, which calculates attention scores for object queries and keys (*Q_i_*, *K_i_*) (*Q_i_*, *K_i_*) (*Q_i_*, *K_i_*):

(12) $$Attention\left(Q_{i},K_{i}\right)=Softmax\left(\frac{Q_{i}K_{i}^{T}}{\sqrt{d_{k}}}\right)\cdot V,$$

While the self-attention mechanism captures global context, it requires a fixed number of object queries, which can limit effectiveness in variable-object scenarios. Despite streamlining the training pipeline, the increased complexity and computational demands may hinder real-time application usability, particularly due to the slower inference times associated with the DC5-R101 backbone. Integrating a CNN module for estimating optimal pooling parameters represents a significant advancement in preprocessing SAR images for ship detection. By leveraging the relationship between LSF values and pooling configurations, this approach optimizes the preprocessing pipeline, ultimately improving detection accuracy in complex maritime environments. Striking a balance between edge sharpness and image fidelity allows the SAR-EDT model to detect ships more effectively amidst clutter and noise, thereby enhancing its utility in real-world applications.

### 2.3. Fusion Non-Maximum Suppression (F-NMS) Module

This research introduces a new fusion framework to improve object detection by combining SAR and optical imagery, particularly in challenging offshore environments. The goal is to align and merge bounding boxes detected in both types of imagery. To achieve this, we match bounding boxes based on the intersection over union (IoU) metric, which is calculated as follows:

(13) $$IoU=\frac{\;\mathrm{Area}\;\mathrm{of}\;\mathrm{Intersection}\;}{\;\mathrm{Area}\;\mathrm{of}\;\mathrm{Union}\;}=\frac{B_{\mathrm{SAR}\;}\cap B_{\mathrm{Optical}}}{B_{\mathrm{SAR}\;}\cup B_{\mathrm{Optical}\;}}$$

where $B_{\mathrm{SAR}\;}$ and $B_{\mathrm{Optical}\;}$ represent the bounding boxes detected in SAR and optical imagery, respectively. Bounding boxes with IOU values exceeding a predefined threshold T are classified as matching, indicating corresponding detections across both modalities.

For these matched bounding boxes, we compute a fused bounding box $B_{\mathrm{Fused}}$ by averaging the center coordinates (x,y) and dimensions (w,h) of the bounding boxes from each modality. The calculation for the fused bounding box is given by the following:

(14) $$\;\;\;w_{\mathrm{Fused}}=\frac{w_{\mathrm{SAR}\;}+w_{\mathrm{Optical}\;}}{2}$$

(15) $$\;\;\;h_{\mathrm{Fused}\;}=\frac{h_{\mathrm{SAR}\;}+h_{\mathrm{Optical}\;}}{2}$$

This averaging approach ensures that the fused bounding box accurately represents the object’s location, thereby minimizing localization errors that can arise from modality-specific variations. For bounding boxes that do not meet the IOU threshold T, each box is preserved independently, thereby ensuring that potentially relevant detections are not overlapping during the fusion process.

To further refine the fused detections, non-maximum suppression (NMS) was applied to eliminate redundant or closely spaced bounding boxes based on their spatial proximity. To determine whether two bounding boxes should be merged, we compared the distance d between their centers with a predefined threshold D.

If d<D, then merge the bounding boxes $B_{i}$ and $B_{j}$. This distance d can be adjusted based on the shift S between the optical and SAR images for the same region, calculated as follows:

(16) $$S=(S_{x},\;S_{y})$$

where $S_{z}$ and $S_{y}$ represent the horizontal and vertical shifts, respectively. The adjusted coordinates of the bounding box centers can then be represented as follows:

(17) $${\bar{x}}_{i}=x_{i}+S_{x}$$

(18) $${\bar{y}}_{i}=y_{i}+S_{y}$$

(19) $${\bar{x}}_{j}=x_{j}+S_{x}$$

(20) $${\bar{y}}_{j}=y_{j}+S_{y}$$

With the adjusted coordinates, the new distance can be recalculated as follows:

(21) $$\bar{d}=\sqrt{{\left({\bar{x}}_{j}-{\bar{x}}_{i}\right)}^{2}+{\left({\bar{y}}_{i}-{\bar{y}}_{j}\right)}^{2}}$$

Finally, the merging conditions are updated. If $\bar{d}<D$, then merge the bounding boxes $B_{i}$ and $B_{j}$.

This method ensures that only relevant bounding boxes are kept, reducing redundancy and improving the precision of the fused detections. By incorporating shift adjustment, this accounts for any misalignment between the SAR and optical images, leading to more accurate and reliable fusion results.

### 2.4. Materials

#### 2.4.1. Datasets

The datasets used in this study are carefully selected to encompass a wide range of scenarios and challenges associated with ship detection in both optical and SAR imagery. These datasets enable the robust training and evaluation of the proposed framework by providing diverse maritime environments, vessel types, and imaging conditions.

The Sentinel-2 Ship (S2) Detection dataset comprises 739 annotated multispectral satellite images captured by ESA’s Sentinel-2 satellites, specifically designed for ship detection in maritime environments. Each image has a resolution of 10 m and is labeled with bounding boxes around ship instances, making it ideal for training and fine-tuning the YOLOv7 object detection model. The dataset includes a diverse range of vessel types, such as small fishing boats, large commercial vessels, cargo ships, and aircraft carriers. It encompasses various maritime scenarios, including islands, lagoons, and deep-sea environments, as well as ships moored in harbors and those traveling side by side. Additionally, some instances are partially obscured by clouds. This diversity in ship types and complex backgrounds ensures robust ship detection across varied environmental conditions, supporting applications requiring precise vessel localization in challenging maritime contexts.

In this study, the SSDD dataset was utilized to train the DETR model for ship detection, while testing was conducted using the SAR-Ship-Dataset. The SAR-EDT module was also trained with test data from both the SSDD and SAR-Ship-Dataset [21], allowing it to leverage a wide range of features across these datasets. This approach enhances detection performance in complex scenes, significantly boosting accuracy and robustness, especially for detecting small ships in challenging conditions. Further details about the SSDD and SAR-Ship-Dataset are provided below, and their key characteristics are outlined in Table 1.

The SSDD dataset comprises 1160 images containing a total of 2456 labeled ship targets, averaging around 2.12 ships per image. As a leading resource in SAR target detection, it includes images from three satellite sensors across four polarization modes, with resolutions ranging from 1 to 15 m. The dataset covers both nearshore and offshore regions, and annotations were created using the LabelImg v.1.8.6 tool, which enhances annotation precision. Using the SSDD adds depth to our study and reinforces the credibility of our findings.

The SAR-Ship-Dataset includes 102 Gaofen-3 and 108 Sentinel-1 images, comprising a total of 43,819 ship slices. Each image has a resolution of 256 × 256 pixels and depicts ships in various scales and backgrounds, enhancing the model’s robustness and adaptability. The dataset is split into training, validation, and test sets in a 7:2:1 ratio, providing a broad feature set for the detection model and contributing to higher accuracy.

Training was conducted on a system equipped with a 12th Gen Intel(R) Core(TM) i7-12700H processor at 2.70 GHz, 16 GB of RAM (15.7 GB usable), and a GeForce RTX 3070 GPU, utilizing the PyTorch v.2.4 deep learning framework.

#### 2.4.2. SAR and Optical Sentinel Satellite Imagery

To enhance the robustness and quality of the ship-specific interpretation dataset, we selected four Level 1 Sentinel-1(S1) Interferometric Wide (IW) swath mode images as the primary data source. The IW mode captures images using Terrain Observation with Progressive Scans SAR (TOPSAR), producing both VV (co-polarization) and VH (cross-polarization) channels. VH cross-polarization offers higher energy intensity, improving the visibility of ship shapes, though it is more susceptible to inshore scattering and sea clutter noise.

Complementing the SAR imagery, we also downloaded Sentinel-2 optical images for the same regions, which include major ports and busy maritime areas, particularly the Suez Canal. This combination captures a diverse range of ship types and complex scenes, facilitating effective ship detection in various environmental conditions.

For image labeling, we utilized the open-source software LabelImg v.1.8.6, allowing for the precise annotation of both SAR and optical images. The images were sourced from archives around March 2021, coinciding with the significant Suez Canal blockage incident involving the Ever Green [22], which stranded at least 369 ships. By synchronizing the acquisition of SAR and optical images during this critical period, the images effectively reflect the maritime situation, supporting the accurate localization of vessels in challenging environments, as in Table 2.

## 3. Results

### 3.1. Experiments of Detection Models on Datasets

In evaluating the performance of YOLOv7 and the DETR on SAR and optical datasets (S1 and S2, respectively), as in Table 3, clear differences emerged in their capabilities across imaging types. YOLOv7 achieved an mAP0.5 of 0.801 and mAP0.5:0.95 of 0.362 on S1 (SAR), demonstrating reliable detection in SAR images but with some limitations in precision across varying IoU thresholds. On S2 (optical), YOLOv7 performed better, with an mAP0.5 of 0.88 and mAP0.5:0.95 of 0.555, reflecting stronger, more consistent detection in optical data. The DETR, however, showed exceptional performance on S1, with an mAP0.5 of 0.93 and mAP0.5:0.95 of 0.53, highlighting its suitability for SAR-based object detection tasks. However, the DETR’s performance dropped significantly on S2, achieving only an mAP0.5 of 0.1, indicating challenges in adapting to optical imagery within this dataset. These results suggest that YOLOv7 is adaptable across both SAR and optical domains, while the DETR excels specifically in SAR detection, making it particularly valuable for SAR-focused applications.

The results clearly show that the DETR performs effectively for ship detection in SAR images, with high accuracy and robustness across various conditions and object sizes, making it well suited for maritime surveillance. However, the DETR’s performance declines for small- and medium-sized ships, particularly in complex environments within SSDD and diverse SAR datasets (Gaofen-3 and Sentinel-1) with varying polarizations and resolutions. This variability highlights areas where the DETR could be refined for the better handling of complex maritime conditions.

**Figure 3:**
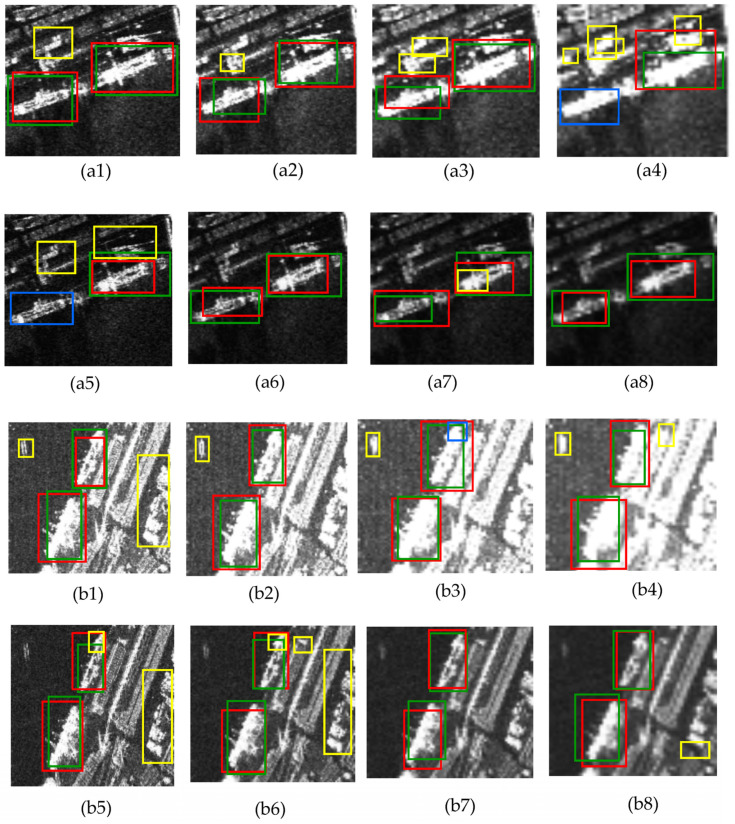
The performance evaluation of the SAR-EDT model on two test datasets: SSDD and SAR-Ship, using SAR images with varying polarizations and resolutions. Panel (**a**) displays the results for maximum pooling (1–4) and median pooling (5–8) with kernel sizes of 3, 5, 7, and 9. Panel (**b**) presents similar results for an image from the SSDD dataset, showing detection outcomes for both pooling methods. The ground truths are marked with green boxes, correct detections with red boxes, missed detections with yellow boxes, and false detections with blue boxes. This figure highlights the effects of different pooling configurations on ship detection performance.

To overcome the limitations observed in the DETR’s performance, especially for smaller ships in complex environments, the SAR-EDT module was introduced. This module incorporates a Convolutional Neural Network (CNN) combined with robust preprocessing techniques to improve feature extraction for the DETR model. This approach was evaluated using the challenging SSDD and SAR ship datasets. As demonstrated in Figure 3, the impact of maximum and median pooling, with kernel sizes of 3, 5, 7, and 9, was tested. For the test images in panels a1-a6, median pooling with a kernel size of 5 yielded the best results, while kernel size 7 was optimal for images in panels b1-6. To further optimize the pooling configurations, metrics such as LSF, PSNR, and F1 score were integrated, enabling the SAR-EDT model to accurately predict the optimal pooling type and kernel size. The model achieved a high prediction accuracy of 91% for pooling type and 92% for kernel size, demonstrating its effectiveness. This approach significantly enhances extraction and detection accuracy, improving the robustness of ship detection in challenging scenarios with varying conditions.

### 3.2. Ablation Study

The ablation experiment evaluated the SAR-EDT model’s performance on the SSD and SAR-Ship datasets. Using the DETR as the baseline for SAR images and YOLOv7 for optical satellite images, this study included Sentinel-1 SAR and Sentinel-2 optical images. Four diverse images were selected, showing ships of various sizes (large, medium, and small) in both offshore and nearshore settings. This setup enabled a balanced examination of the detection methods under realistic and varied environmental conditions. SAR-EDT was applied to SAR images and YOLOv7 to optical images, with the fusion framework added to enhance detection across both models. This configuration allowed for a comprehensive assessment of the proposed method’s effectiveness in improving detection accuracy across different ship sizes and complex environments.

For the quantitative analysis of ship detection performance, we evaluate precision (*P*), recall (*R*), and F1 score (F1) as the key metrics. These indicators are defined as follows:

(22) $$P=\frac{TP}{TP+FP}$$

(23) $$R=\frac{TP}{TP+FN}$$

(24) $$F1=\frac{2\times P\times R}{P+R}$$

where *TP*, *FP*, and *FN* represent True Positives, false positives, and False Negatives, respectively. For ships with wakes, a detection is considered a True Positive if the head endpoint is within three pixels of the ground truth. For ships without wakes, the detection is classified as a True Positive if the center point between the two endpoints is within three pixels of the ground truth.

The results showed that SAR-EDT improved significantly over the DETR baseline in SAR images due to its integrated CNN preprocessing module, which enhanced feature extraction, especially for detecting small ships. Some detection challenges persisted in specific onshore locations, however, indicating areas for further optimization. YOLOv7 performed well on optical images under clear conditions but struggled in cloudy scenarios where visual clarity was reduced. Here, the fusion module played a crucial role, compensating for these limitations and bolstering detection accuracy for smaller targets, particularly in challenging weather.

The comparison of SAR, optical, and fused detection methods across various conditions consistently highlights the advantages of SAR–optical fusion, particularly under challenging weather conditions where single modalities face limitations, as shown in Table 4. In clear onshore environments, SAR achieved 100% precision, 90% recall, and a 95% F1 score, while optical imagery achieved perfect scores across all metrics. The combination of SAR and optical data further improved accuracy, with the fusion method reaching 99% in precision, recall, and F1 score. Under cloudy onshore conditions, SAR maintained a high recall of 100%, though precision dropped to 80%, resulting in an F1 score of 88.9%. Optical imagery performed poorly in these conditions with 0% in all metrics, but the fusion method mirrored SAR’s performance, matching its precision, recall, and F1 score. In clear offshore conditions, both SAR and optical methods achieved 99% precision, 91% recall, and a 95% F1 score individually, while the fusion method again produced perfect results. In offshore cloudy conditions, SAR maintained 99% precision but experienced a slight decrease in recall to 81%, leading to a 90% F1 score, whereas optical imagery achieved 99% precision, 91% recall, and a 95% F1 score. Fusion outperformed both individual modalities in this case, achieving 99% precision, 97% recall, and a 98% F1 score.

**Figure 4:**
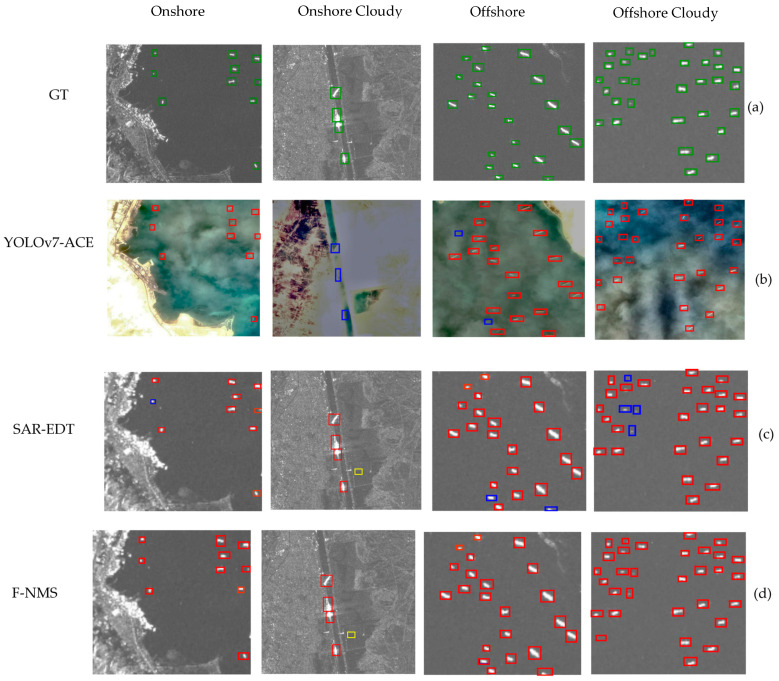
This figure presents the experimental results for ship detection in SAR images across four distinct regions: onshore, onshore cloudy, offshore, and offshore cloudy. Subfigure (**a**) displays the ground truth images, while subfigures (**b**–**d**) illustrate the detection results from the YOLOv7-ACE model, SAR-EDT with optimized pooling and kernel size, and the F-NMS method. In these subfigures, ground truths are highlighted with green boxes, predicted detections are marked with red boxes, false detections are indicated with yellow boxes, and missed detections are represented by blue boxes.

These results underscore the effectiveness of SAR–optical fusion, particularly in complex or cloudy conditions, where it mitigates YOLOv7′s vulnerability to cloud cover and compensates for SAR’s challenges in maintaining consistent accuracy. By combining both modules, the fusion approach enhances detection reliability and accuracy across a wide range of environments, offering more robust performance under variable weather and lighting conditions. As illustrated in Figure 4, this fusion method proves to be especially beneficial in improving detection outcomes when faced with difficult environmental factors.

## 4. Discussion

### 4.1. Implementation Details

This study combines advanced preprocessing, data augmentation, and a novel two-stage detection framework to improve object detection across SAR Sentinel-1 and optical Sentinel-2 satellite datasets. Preprocessing techniques included auto-orienting pixel data and resizing images to 500 × 500 pixels, ensuring uniformity and computational efficiency. Augmentation strategies, such as random rotations (−15° to +15°), horizontal and vertical flips (50% probability), brightness adjustments (−25% to +25%), and salt-and-pepper noise applied to 1% of pixels, were employed to simulate diverse real-world conditions like varying angles, lighting, and sensor noise.

YOLOv7 and the DETR were trained and evaluated across these datasets. YOLOv7 demonstrated robust performance, requiring 50 epochs over 12 h for SAR data and 2000 epochs over 72 h for higher-resolution optical data. The DETR excelled in SAR image detection, completing training in 8 h over 50 epochs, leveraging its Transformer-based architecture for effective feature extraction. However, the DETR struggled with low-resolution Sentinel-2 optical images, failing to produce meaningful results despite training for 100 to 150 epochs.

The detection framework further integrates a CNN to refine input features before feeding them into the DETR. In this second stage, the CNN optimizes parameters such as kernel size and pooling type to enhance detection performance. It is trained using the Adam optimizer (learning rate: 0.001) and Cross-Entropy Loss (CEL) over 100 epochs. The CEL function is defined as follows:

(25) $$CEL=-\frac{1}{N}\sum_{i=1}^{N}\;y_{i}log\;\left({\hat{y}}_{i}\right)+\left(1-y_{i}\right)log\;\left(1-{\hat{y}}_{i}\right)$$

where *N* represents the total number of samples, $y_{i}$ is the true label (either 0 or 1 for binary classification), and $\hat{y}$ is the predicted probability of the positive class. The first term, $\;y_{i}log\;\left({\hat{y}}_{i}\right)$, penalizes incorrect predictions for positive samples, while the second term, $\left(1-y_{i}\right)log\;\left(1-{\hat{y}}_{i}\right)$, penalizes incorrect predictions for negative samples. By minimizing CEL, the model adjusts its parameters to ensure higher probabilities for true classes, thereby improving prediction accuracy.

This comprehensive two-stage approach effectively combines the DETR for precise detection and the CNN for feature optimization, significantly enhancing detection accuracy in complex SAR environments. By tailoring preprocessing, augmentation, and model-specific training strategies, this study addresses the challenges posed by diverse satellite imagery, balancing resolution requirements and computational efficiency.

### 4.2. The Effectiveness of Preprocessing CLAHE for Yolov7

Applying CLAHE as a preprocessing step on S2 optical images has proven highly effective in enhancing image quality before using the YOLOv7 detection model. This study applied CLAHE to four different regions: onshore, onshore cloudy, offshore, and offshore cloudy.

For onshore regions, the original histogram exhibited pixel intensities clustered between 100 and 230, with a sharp peak near 255, indicating overexposure and the underrepresentation of darker regions (intensities below 100). CLAHE redistributed the intensities more evenly across the 0–255 range, significantly reducing the sharp peak near 255 and improving the visibility in shadowed and mid-tone areas. This resulted in enhanced local contrast, reducing overexposure and ensuring a better representation of both dark and bright regions.

For onshore cloudy regions, the original pixel intensities were heavily concentrated in a narrow range (150–160), with a peak frequency exceeding 100,000, leading to a washed-out appearance with poor contrast. After applying CLAHE, the intensity values were redistributed across the entire 0–255 range, lowering the peak frequency to approximately 30,000. This adjustment improved the visibility of darker and brighter regions, significantly enhancing the overall contrast and image clarity. The redistribution of pixel values ensured a better differentiation of features, making the image more suitable for downstream analysis.

For offshore regions, the original image had over 600,000 pixels clustered near 250, signifying extreme overexposure and low contrast. CLAHE effectively redistributed the intensities, lowering the peak frequency to approximately 14,000 while enhancing visibility in darker regions and improving balance throughout the image.

In offshore cloudy regions, the original histogram showed intensities concentrated between 50 and 150, with prominent peaks near 100, indicating poor brightness variation and limited dynamic range. After CLAHE, the peak frequency dropped from over 7000 to approximately 5000, and the intensity distribution smoothed across the full range, expanding the dynamic range. This resulted in the better visibility of darker and brighter areas and enhanced image details.

Overall, CLAHE enhanced contrast, balanced pixel intensities, and improved details in all four scenarios, demonstrating its effectiveness in preprocessing S2 images for improved object detection with the YOLOv7 model.

#### SAR-EDT Module Effect Validation

In this section, the analysis focuses on the performance of SAR-EDT on VV and VH polarizations across four different regions, predicting the optimal pooling method (max or median) and kernel size for each region. By calculating the mean and standard deviation of pixel intensity values for both VV and VH images, we assess scattering behavior and noise levels, which inform the selection of the most suitable pooling method.

**Figure 5:**
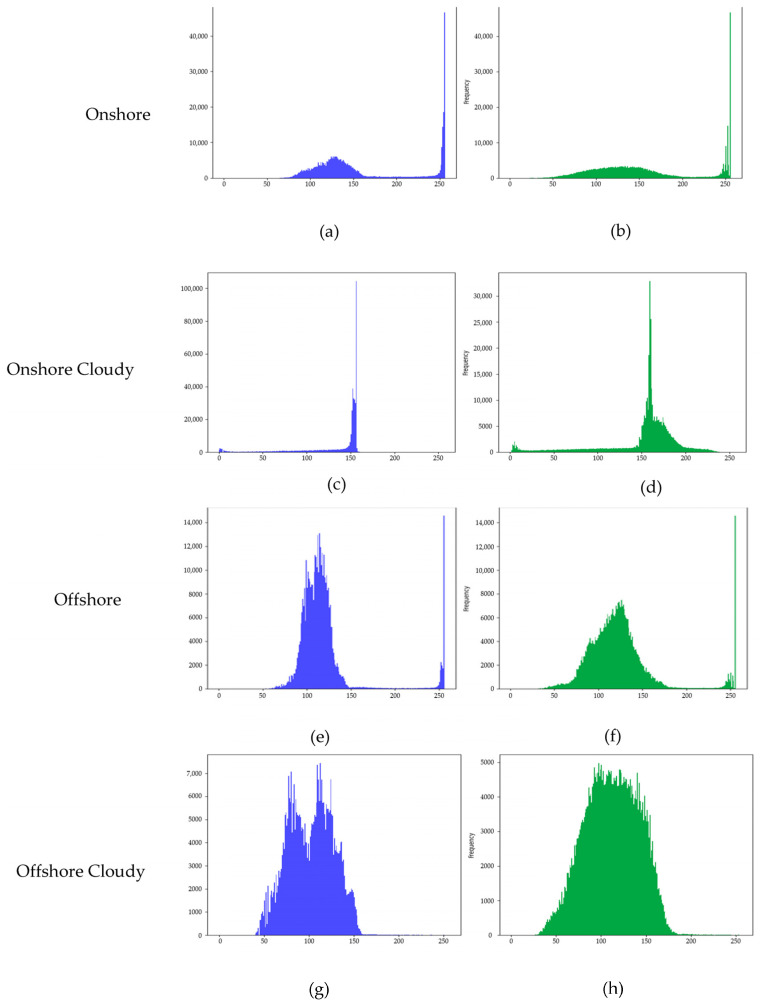
The impact of Contrast Limited Adaptive Histogram Equalization (CLAHE) on Sentinel-2 images under four scenarios: onshore (**a**,**b**), onshore cloudy (**c**,**d**), offshore (**e**,**f**), and offshore cloudy (**g**,**h**). Subfigures show the pixel intensity distribution before (**a**,**c**,**e**,**g**) and after (**b**,**d**,**f**,**h**) applying CLAHE. The *x*-axis represents pixel intensity, and the *y*-axis represents frequency.

**Figure 6:**
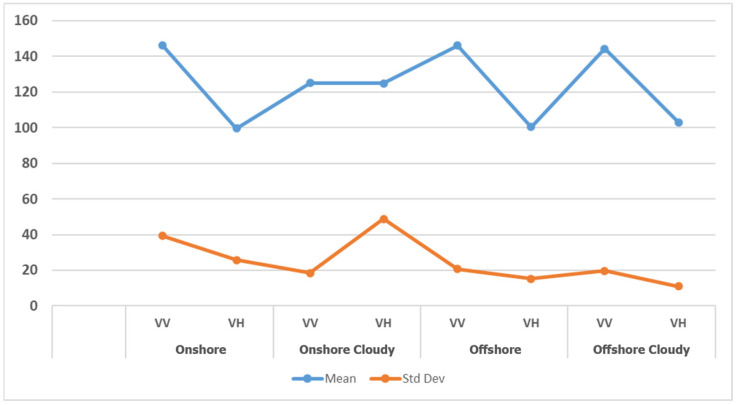
The mean and standard deviation values for various polarization images in the four regions.

The results, summarized in Figure 5 and Figure 6, show that in the first scenario (onshore), VV images exhibit higher backscattering but lower noise levels in VH, making VH images with max pooling and a kernel size of 5 the best choice for ship detection. In the second scenario (onshore cloudy), while VV images have more backscattering, median pooling with a kernel size of 7 on VV images provides better performance by reducing noise. In the third scenario (offshore), although VV backscattering is higher, VH images with max pooling and a kernel size of 5 outperform VV images due to lower noise levels. Finally, in the fourth scenario (offshore cloudy), VV images again show more backscattering, but VH images with max pooling and a kernel size of 5 yield the best detection performance due to minimal noise.

**Figure 7:**
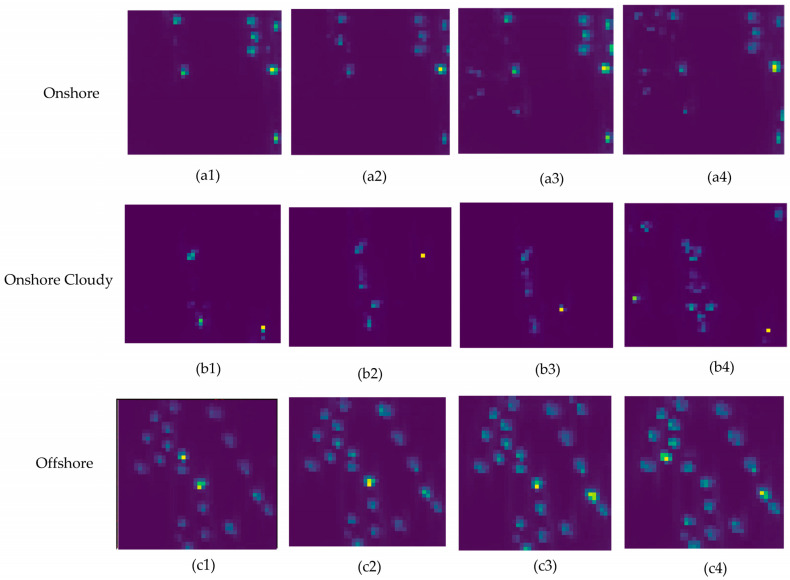

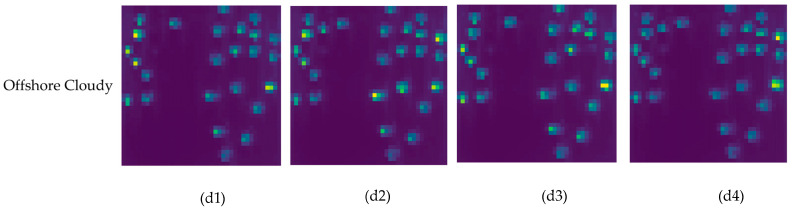
Attention maps showcasing the impact of pooling methods and kernel sizes (3 × 3, 5 × 5, 7 × 7, 9 × 9). The maps illustrate: (**a1**–**a4**) max pooling results for onshore VH images, (**b1**–**b4**) median pooling results for onshore cloudy VV images, (**c1**–**c4**) max pooling results for offshore VH images, and (**d1**–**d4**) max pooling results for offshore cloudy VH images. These visualizations demonstrate the SAR-EDT model’s effectiveness in enhancing detection accuracy.

These results highlight the strength of our fusion approach, where SAR-EDT effectively selects median pooling for VV and max pooling for VH to optimize detection performance. The attention maps in Figure 7 demonstrate how the preprocessed CNN pooling modules enhance detection by applying the optimal kernel sizes and pooling methods (median for VV and max for VH), showing a clear improvement in detection across all regions. This validates the effectiveness of our approach in refining detection accuracy in various SAR imaging conditions.

The limitations of our research include the reliance on high-quality optical imagery, which can be compromised in adverse weather conditions like rain or fog, affecting detection accuracy. Although the SAR-EDT model uses denoising techniques, it may not completely eliminate noise in environments with low signal-to-noise ratios. Additionally, the fusion process may struggle with overlapping or congested objects, potentially leading to missed detections or false positives. The effectiveness of the framework depends on the optimal selection of pooling parameters, which might not have good generalizability across all conditions. Finally, real-time processing and scalability could be challenging due to the computational complexity of the fusion and postprocessing steps.

## 5. Conclusions

This research introduces a novel fusion framework that integrates optical and synthetic aperture radar (SAR) imagery to enhance ship detection accuracy. By leveraging the complementary strengths of optical and SAR data, this framework addresses challenges associated with individual modalities, such as weather conditions, noise levels, and geometric misalignment between images from heterogeneous sensors. Optical imagery, enhanced through Contrast Limited Adaptive Histogram Equalization (CLAHE) and processed via the YOLOv7 model, provides detailed visual information. Meanwhile, the customized SAR-EDT model improves ship detection in SAR imagery, particularly under adverse conditions. The fusion module combines detection outputs using intersection over union (IoU) and confidence scores, further refining the results and improving detection reliability and precision. This integrated approach effectively mitigates the limitations of individual modalities and compensates for geometric misalignment between images from different sensors. Despite the challenge of real-time processing, the framework shows significant potential in improving ship detection in dynamic and complex maritime environments. Future work can focus on optimizing these aspects, expanding the framework’s applicability to a broader range of scenarios, and enhancing computational efficiency to support real-time deployment.

## Figures and Tables

**Table 1:** Characteristics and specifications of ship detection datasets.

Datasets	Res. (m)	Band	Res. (m)	Satellites	Polarization
SSDD	1∼15 m	C/X	1∼15 m	S-1, RadarSat-2, TerraSAR-X	HH, VV, VH, HV
SAR-Ship-Dataset	3∼25 m	C	3∼25 m	S-1, GF-3	HH, VV, VH, HV

**Table 2:** Attributes of SAR–optical data used in this study.

Parameters	Sentinel-1B	Sentinel-2A
Product Type	GRD	Level 1C
Polarization	VV-VH	__
Mode	IW	__
Band	C-Band (5.405 GHz)	Band 2: 496.6 ± 98 nm Band 3: 560.0 ± 46 nm Band 4: 664.5 ± 39 nm
Spatial Resolution	10 m	10 m
Acquisition Date	24 Mar 2021	24 Mar2021
Location	Egypt—Suez Canal	Egypt—Suez Canal

**Table 3:** Performance analysis of YOLOv7 and DETR on optical and SAR datasets using mean average precision.

Model	Data	AP 50:95 (%)	AP 50 (%)
Yolov7	SAR	362	80
	Optical	55	88
DETR	SAR	53	93
	Optical	0	1

**Table 4:** Performance evaluation of ship detection in SAR and optical imagery across onshore and offshore environments.

Area	Method	Image	Precision %	Recall %	F1 Score %
Onshore	SAR-EDT	SAR	98	90	95
	YOLOv7-ACE	Optical	98	98	98
	F-NMS	Optical + SAR	99	99	99
Onshore Cloudy	SAR-EDT	SAR	80	99	88.9
	YOLOv7-ACE	Optical	0	0	0
	F-NMS	Optical + SAR	80	99	88.9
Offshore	SAR-EDT	SAR	99	91	95
	YOLOv7-ACE	Optical	99	98	95
	F-NMS	Optical + SAR	99	98	99
Offshore Cloudy	SAR-EDT	SAR	99	81	90
	YOLOv7-ACE	Optical	98	91	95
	F-NMS	Optical + SAR	99	97	98

## Data Availability

Data are contained within this article.
